# Novel Nested Peptide Epitopes Recognized by CD4^+^ T Cells Induced by HIV-1 Conserved-Region Vaccines

**DOI:** 10.3390/vaccines8010028

**Published:** 2020-01-16

**Authors:** Nicola Borthwick, Sandra Silva-Arrieta, Anuska Llano, Masafumi Takiguchi, Christian Brander, Tomáš Hanke

**Affiliations:** 1 The Jenner Institute, University of Oxford, Oxford OX3 7DQ, UK; nicola.borthwick@ndm.ox.ac.uk; 2 IrsiCaixa AIDS Research Institute-HIVACAT, Hospital Universitari Germans Trias i Pujol, 08916 Badalona, Barcelona, Spain; ssilva@irsicaixa.es (S.S.-A.); ALlano@irsicaixa.es (A.L.); cbrander@irsicaixa.es (C.B.); 3 Joint Research Center for Human Retrovirus Infection, Kumamoto University, Kumamoto 860-0811, Japan; masafumi@kumamoto-u.ac.jp; 4 Faculty of Medicine, Universitat de Vic-Central de Catalunya (UVic-UCC), 08500 Vic, Spain; 5 Institució Catalana de Recerca I Estudis Avançats (ICREA), 08010 Barcelona, Spain

**Keywords:** HIV vaccine, HIVconsv, conserved regions, CD4 epitopes, HLA class II epitopes, T cell vaccine

## Abstract

CD4^+^ T-cell responses play an important role in the immune control of the human immunodeficiency virus type 1 (HIV-1) infection and as such should be efficiently induced by vaccination. It follows that definition of HIV-1-derived peptides recognized by CD4^+^ T cells in association with HLA class II molecules will guide vaccine development. Here, we have characterized the fine specificity of CD4^+^ T cells elicited in human recipients of a candidate vaccine delivering conserved regions of HIV-1 proteins designated HIVconsv. The majority of these 19 most immunogenic regions contained novel epitopes, that is, epitopes not listed in the Los Alamos National Laboratory HIV Sequence Database, which were able in vitro to stimulate vaccinees’ CD4^+^ T cells to proliferate and produce interferon-γ and tumor necrosis factor-α. Accumulation of HLA class II epitopes will eventually accelerate development of HIV-1 prophylactic and therapeutic vaccines.

## 1. Introduction

There is no doubt that T cells are an important component of the host immune defense against HIV-1 infection [1,2]. Adaptive cellular antiviral responses are mediated by CD8^+^ and CD4^+^ T cells, which recognize foreign peptides presented by self-major histocompatibility complex molecules, in humans designated human leukocyte antigens (HLA), of class I and II, respectively. Upon stimulation, CD8^+^ and CD4^+^ T cells produce a variety of soluble intercellular signaling molecules leading to cell death and inducing an antiviral state in cells in their immediate vicinity. The convention is to regard CD8^+^ T cells as the effector cells killing HIV-1-infected cells and CD4^+^ T cells as the helper cells to both CD8^+^ cytolytic cells and B cells producing antibodies, however, it is well documented that CD4^+^ T cells can also exert cytolytic activity [3,4,5,6,7]. Both CD8^+^ [8,9,10,11] and CD4^+^ [5,6,12,13,14,15,16] T-cell responses have been associated with HIV-1 immune control and their broadly specific coordinated actions are likely to be important for protection.

CD8^+^ T cells are highly selective for peptides presented by HLA class I molecules, which has allowed for a relatively accurate experimental definition of optimal peptide lengths (usually 8 to 10 amino acids) and HLA-binding motifs [17,18]. In contrast, definition of common patterns for HLA-class II-associated peptides has been more challenging [19,20,21]. HLA class II binds nested peptides of up to 20 amino acids, which consist of a core sequence with anchor residues typically at positions P1, P4, P6, and P9 [22] surrounded by weakly interacting flanking region sequences (FRSs), which are embedded within the HLA binding groove and influence the binding affinity between the peptide and HLA molecule as well as recognition of HLA–peptide complexes by the T-cell receptor (TCR) [23,24,25,26,27,28,29,30]. Naturally processed HLA class II-bound peptides have FRSs at both N- and C-termini and these are of variable lengths. Furthermore, epitopes can often be overlapping and class II restricting molecules are promiscuous; that is, a single class II molecule can bind multiple peptides and a single peptide can be presented by several different class II molecules [31]. Previous studies suggested that the majority of HLA class I and II molecules can be grouped into broad supertypes binding cross-reactive peptides bearing common supermotifs [21,32]. Studies of specific epitopes suggested that the peptide length and sequence variation (altered peptide ligands) can determine whether a peptide elicits a full (agonist) or partial (partial agonist) activation phenotype or, in fact, inhibits (antagonist) the CD4^+^ T-cell response to a stimulatory peptide [33,34,35,36,37,38]. Thus, careful characterization of the HLA class I- and class II-associated epitopes derived from the HIV-1 proteome will critically guide development of an effective vaccine. For this very reason, HLA class I and II HIV-1 epitopes are collected, regularly updated, and published by curators of the Los Alamos National Laboratory HIV Sequence Data [39].

With the aim to counteract HIV-1 sequence variability and induce truly protective T-cell responses, we constructed a candidate T-cell vaccine delivering chimeric immunogen HIVconsv, which consists of segments of 14 highly conserved regions of HIV-1 proteins Gag, Pol, Vif, and Env [40]. Each region employed a clade consensus amino acid sequence and the four major HIV-1 clades A, B, C, and D were alternated to provide equal clade representation. Because T-cell responses to conserved HIV-1 regions are often subdominant in natural infection, their delivery out of the context of the whole virus by a potent vaccine regimen revealed novel T-cell determinants [41,42,43]. We previously described the definition of novel CD8^+^ T-cell epitopes induced in the HIVconsv vaccine recipients [41,42]. Here, in the same human volunteers, we characterize the peptide epitopes recognized by vaccine-elicited CD4^+^ T cells.

## 2. Materials and Methods

### 2.1. Ttial HIV-CORE 002l

The HIV-CORE 002 trial [1,44,45] recruited healthy adults of low risk of HIV-1 infection in Oxford UK in 2011–2012. The trial was approved by the National Research Ethics Service (NRES) Committee West London (Ref: 10/H0707/52) and the Medicines and Healthcare products Regulatory Agency of the UK (Ref: 21584/0271/001). The study was carried out according to the principles of the Declaration of Helsinki (2008) and complied with the guidelines of the International Conference on Harmonization Good Clinical Practice. All volunteers gave written informed consent prior to participation. Samples of the peripheral blood mononuclear cell (PBMC) are kept in the Oxford Vaccine Centre Biobank in compliance with the UK Human Tissue Act 2004 approved by NRES (Ref: 10/H0504/25).

### 2.2. HLA Typing

Tissue typing for the HLA-DRB molecules was performed by The DNA Sequencing MISEQ HLA Laboratory, Weatherall Institute of Molecular Medicine, The John Radcliffe, on EDTA blood samples collected prior to vaccination. Genomic DNA was isolated using purification kit ArchivePure^TM^ DNA (5 PRIME GmbH) and the tissue types were determined using oligonucleotide probes in SS-PCR.

### 2.3. Peptides

Individual HIVconsv 15-mer peptides overlapping by 11 amino acids as well as truncated peptides were >90% pure (GenScriptHK, Hong Kong) and were dissolved in DMSO to 10–40 mg/mL, diluted in PBS to working stock solutions of 4 mg/mL and used at final concentrations of 1.5 µg/mL. Terminal lysine (K) was added to some 15-mer peptides for solubility.

### 2.4. Short-Term Cell Lines (STCL)

Cryopreserved PBMC samples were thawed and cultured at 1–3 × 10^6^ cells/mL in the R10 medium (RPMI 1460 supplemented with 10% FBS, 2 mM L-glutamine, 1 mM sodium pyruvate, 10 mM HEPES, and penicillin–streptomycin antibiotics; Sigma Aldrich, Poole, UK) containing either a 15-mer peptide pool with 1.5 µg/mL of each peptide or just the ‘parental’ 15-mer peptide with added 25 ng/mL of IL-7 (R&D Systems). One hundred IU/mL IL-2 (R&D Systems, Minneapolis, MN, USA) and IL-2 plus fresh culture medium were added on days 3 and 7, respectively. On day 10, cells were washed 3x with R10, resuspended in 1 mL of R10, and rested at 37 °C, 5% CO_2_ for 48 h prior to the ICS assay.

### 2.5. IFN-γ ELISPOT Assay

Cryopreserved PBMCs were tested in an IFN-γ ELISPOT assay as described previously [44]. ELISPOT plates (S5EJ044I10; Merck Millipore, Bedford, MA, USA) were prewetted for 1 min with 15 µL of 35% ethanol and coated with anti-IFN-γ antibody at 10 µg/mL in PBS (clone 1-D1K; Mabtech AB, Nacka Strand, Sweden) at 4 °C for 16 h. Next day, plates were washed with PBS and blocked with R10 at 37 °C for at least 1 h. STCLs were added at 4 × 10^4^ cells/well into triplicate wells for peptide pools and duplicate wells for individual peptides at a final concentration of 1.5 µg/mL each and incubated at 37 °C, 5% CO_2_ overnight. Six no-peptide wells with cells and 0.45% DMSO served as a negative control. Cells cultured with 10 µg/mL PHA (Sigma Aldrich, Poole, UK) served as a positive control. Next day, wells were incubated with biotin-conjugated anti-IFN-γ mAb followed by alkaline phosphate-conjugated streptavidin (both from Mabtech AB) and color substrate BCIP/NBT^Plus^ (Mabtech AB). The reaction was stopped after 5 min by washing with tap water and the plates were air dried. Next day, the spots were counted by an AID ELISpot Reader with version 5.0 software (AID GmbH, Straβberg, Germany). The results were expressed as median net SFU/10^6^ PBMC after subtracting the median number of spot-forming units (SFU) in no-peptide wells from the test wells. Positive responses were defined as at least 50 SFU/10^6^ PBMC above background and at least twice the background of wells without peptide stimulation.

### 2.6. Intracellular Cytokine Staining and Flow Cytometry

Short-term cell line (STCL) cultures were incubated in 96-well plates in R10 with or without a peptide, or with SEB (Staphylococcus enterotoxin B, Sigma-Aldrich) as a positive control, plus anti-CD28 and anti-CD49d mAbs at 1 µg/mL, brefeldin A at 5.0 µg/mL, and Golgi Stop (BD Biosciences, Workingham, UK) at 37 °C, 5% CO_2_ for 6 h and placed at 4 °C overnight. Next morning, cells were incubated with a viability marker (LIVE/DEAD fixable Aqua; Invitrogen, Inchinnan, UK), anti-CD8 FITC-, and anti-CD4 PE-conjugated mAbs (BD Biosciences), permeabilized using Fix/Perm (BD Biosciences), stained with anti-CD3 ECD- (Beckman Coulter, High Wycomb, UK), anti-IFN-γ V450- (BD Biosciences), and anti-TNF-α APC- (BD Biosciences) conjugated mAbs at 4 °C for 30 min and fixed in 1% paraformaldehyde. The cells were acquired on LSR II flow cytometer (Becton-Dickinson, Swindon, UK). Data were analyzed using FlowJo software (Tree Star, Ashland, OR, USA).

The gating strategy was employed as follows: (i) FSC-A vs. FSC-H to gate out doublets, (ii) FSC vs. SSC wide gate to exclude cell debris, (iii) CD3 vs. LIVE/DEAD to gate on viable CD3^+^ T-cells, (iv) CD4 vs. CD8 to gate on single-positive CD4 and CD8 T cells and (v) IFN-γ and TNF-α CD4^+^ subsets.

### 2.7. Epitope Prediction

Peptides identified by the ICS analysis of peptide pool- or individual peptide-expanded STCL and the volunteer’s HLA types were entered into the Epitope Location Finder (ELF) algorithm [46] and compared to previously reported epitopes retrieved from the T-helper/CD4^+^ Epitope Summary [47]. Potential HLA DRB1 restriction was suggested based on the match of anchor residues and motifs associated with the submitted HLAs.

## 3. Results

### 3.1. The Study Subjects and Vaccination

**Figure 1:**
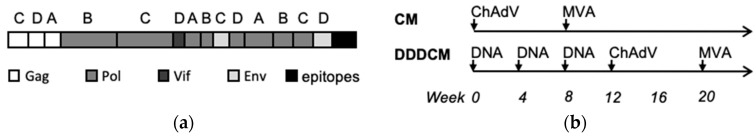
The HIVconsv immunogen and vaccination. (**a**) Schematic representation of the HIVconsv immunogen derived from 14 conserved regions of the HIV-1 proteome. For each segment, the clade of consensus amino acid sequence is shown above and the HIV-1 proteins from which it was derived are color-coded. C-terminal CD8^+^ T-cell and monoclonal antibody epitopes were added to facilitate preclinical vaccine development and manufacture. (**b**) Two vaccination regimens used for the volunteers of this study. ChAdV—ChAdV63.HIVconsv; MVA—MVA.HIVconsv; DNA—pSG2.HIVconsv.

Healthy, HIV-1/2-negative adults in Oxford, UK received the HIVconsv immunogen (Figure 1a) delivered by a combination of plasmid DNA, nonreplicating engineered simian adenovirus of chimpanzee origin ChAdV63, and nonreplicating poxvirus modified vaccinia virus Ankara (MVA) (Figure 1b) [42,44]. Subjects’ HLA genotypes, vaccination regimens, and peak of ex vivo fresh interferon (IFN)-γ ELIPOST assay frequencies of HIV-1-specific T cells (sum of CD8^+^ and CD4^+^) are listed in Table 1.

### 3.2. CD4^+^ T-Cell Epitope Mapping

The primary HIV-CORE 002 immunological readout employed 199 15-mer peptides overlapping by 11 amino acids, which spanned the entire HIVconsv protein. These HC001-HC199 peptides were divided into six pools P1–P6 and used to determine the frequencies of HIVconsv-specific responses in vaccine recipients in a fresh ex vivo IFN-γ ELISPOT assay [44]. For mapping of stimulatory 15-mer peptides, cryopreserved PBMCs collected between 10 weeks and 1 year after the last vaccine administration were first expanded in vitro for 10 days using individual peptide pools to establish a short-term cell line (STCL), which was then tested against each 15-mer of the original pool in an IFN-γ ELISPOT assay. To narrow down optimal peptide length and identify CD4^+^ T cells, single stimulatory 15-mer ‘parental’ peptides used to expand PBMCs for 10 days and the resulting SCTLs were incubated with progressively truncated peptides in an ICS assay and the cytokine production by CD3^+^ CD8^+^ and CD3^+^ CD4^+^ T cells was monitored. For 11 individuals showing positive CD4^+^ T-cell responses, optimal epitope cores were predicted in silico based on the subjects’ HLA class II molecules as well as assessed experimentally. Full subjects’ HLA class I types are given in Table 1, while only the potentially restricting molecules are given in the text. The results are reported below in the order of the 15-mer peptide, in which they appear in the HIVconsv immunogen, and the HIV-1 protein of origin and HXB2 amino acid location are also provided below the peptide schematics.

#### HC003 EVIPMFTALSEGATP (Gag 167–181)

**Figure 2:**
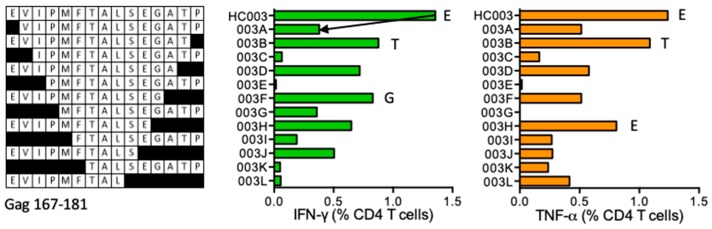
HC003/VID 418—definition of CD4^+^ T-cell epitopes in HIV-1 protein Gag. Cryopreserved PBMC from vaccine recipient 418 were expanded by stimulation with ‘parental’ 15-mer responder peptide for 10 days to establish effector STCL. These were subjected to ICS using serially truncated (left) and a positive CD4^+^ T-cell production of IFN-γ (green) and TNF-α (orange) was monitored. Arrows and amino acids E, T and G indicate the peptide-terminal residues required for efficient peptide recognition.

One subject 418 (DRB1*04:01 DRB1*07:01) developed CD4^+^ T-cell responses to the HC003 peptide with stimulatory shorter peptides EVIPMFTALSEGAT (ET14), EVIPMFTALSEGA (EA13), EVIPMFTALSEG (EG12), EVIPMFTALSE (EE11), and EVIPMFTALS (ES10) (Figure 2). Peptide PMFTALSEGAT is reported in the LANL-HSD without restriction as well as the **P**EVIPMF**S**ALSEGATP peptide, which is restricted by DR1. The Epitope Location Finder (ELF) of the LANL-HSD predicts two epitope cores EVIPMFTALS and FTALSEGAT to bind DRB1*04:01, and VIPMPT and LSEGAT to bind to DRB1*07:01 with the anchor residues underlined.

#### HC017 GLNKIVRMYSPVSIL (Gag 269–283)

**Figure 3:**
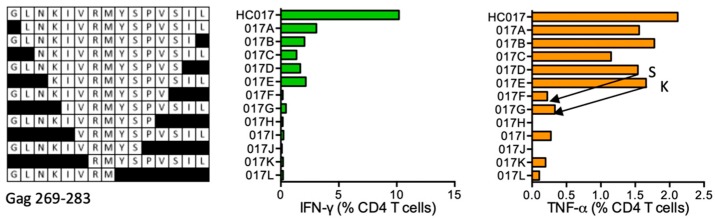
HC017/VID 418—definition of CD4^+^ T-cell epitopes in HIV-1 protein Gag. 15-mer-expanded cryopreserved PBMC of volunteer 418 were stimulated with truncated peptides (left) in ICS and production of IFN-γ (green) and TNF-α (orange) by CD4^+^ T cells was determined. Arrows in the graph and amino acids S and K indicate the stimulatory peptide termini.

CD4^+^ T cells from HC017 STCL of subject 418 (DRB1*04:01 DRB1*07:01) responded to 15-mer peptide GLNKIVRMYSPVSIL and shorter peptides down to GLNKIVRMYSPVS (GS13) and KIVRMYSPVSIL (KL11) were still strongly stimulatory (Figure 3). This 15-mer peptide of clades A and B is reported in the LANL-HSD without identified HLA restriction, while this and shorter peptides with the S281T substitution KIVRMYSP**T**S and KIVRMYSP**T** are reported with DRB1*01:01 and DR4, respectively. NKIVRMYSP**T**SI was reported binding to eight different DRB1 molecules tested including DRB1*01:01 [31]. ELF predicts epitope cores LNKIVRMYS for DRB1*04:01 and IVRMYS for DRB1*07:01.

#### HC078 YFSVPLDEGFRKYTA (Pol 270–284)

**Figure 4:**
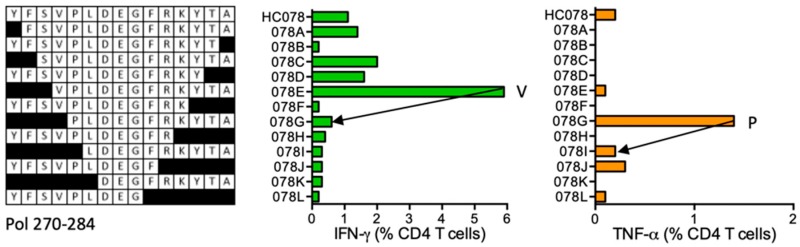
HC078/VID 417—definition of CD4^+^ T-cell epitopes in HIV-1 protein Pol. PBMC of subject 417 were expanded by HC078 and stimulated with truncated peptides (left) in ICS. IFN-γ (green) and TNF-α (orange) producing CD4^+^ T cells were determined. Arrows in the graphs and amino acids V and P indicate the stimulatory peptide termini.

Volunteer 417 (DRB1*01:01 DRB1*07:01) developed CD4^+^ T-cell responses to peptide HC078, shorter peptides and the strongest VPLDEGFRKYTA (VA12) stimulating IFN-γ and relatively weaker peptide PLDEGFRKYTA (PA11), which induced TNF-α (Figure 4). Therefore, there are potentially two different epitopes producing different cytokines and stimulating different CD4^+^ T-cell populations. None of these epitopes is reported in the LANL-HSD nor are these predicted by the ELF for the volunteer’s HLA class II alleles.

#### HC079 PLDEGFRKYTAFTIP (Pol 274–288)

**Figure 5:**
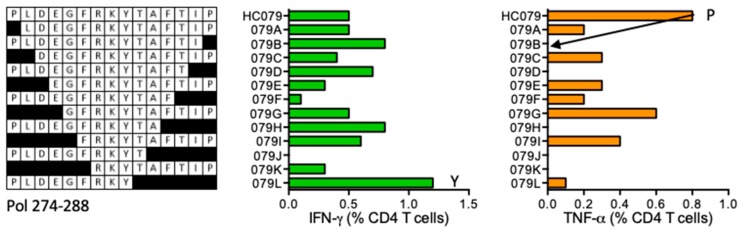
HC079/VID 417—definition of CD4^+^ T-cell epitopes in HIV-1 protein Pol. HC078 STCL was generated using cryopreserved PBMC of volunteer 417 and tested against truncated peptides (left) in ICS. IFN-γ (green) and TNF-α (orange) producing CD4^+^ T cells were enumerated. Arrows and amino acid Y and P indicate the stimulatory peptide termini.

Vaccine recipient 417 (DRB1*01:01 DRB1*07:01) developed CD4^+^ T cells recognizing peptide HC079. The ICS assay confirmed parental 15-mer HC079, and suggested truncated PLDEGFRKY (PY9) and FRKYTAFTIP (FP10) peptides as the minimum epitopes stimulating for IFN-γ and TNF-α production, respectively (Figure 5). These peptides are not reported or predicted to bind volunteer’s DRB1 molecules.

#### HC080 GFRKYTAFTIPSINN (Pol 278–292)

**Figure 6:**
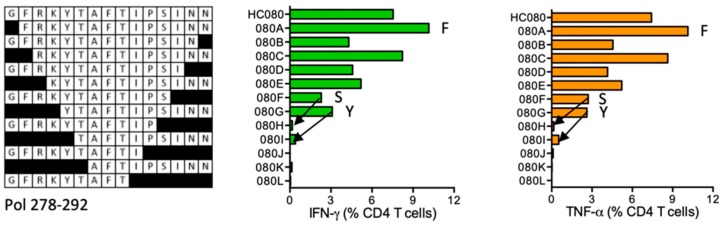
HC080/VID 411—definition of CD4^+^ T-cell epitopes in HIV-1 protein Pol. Expanded PBMCs from volunteer 411 were stimulated with truncated peptides (left) in ICS and the elicited production of IFN-γ (green) and TNF-α (orange) by CD4^+^ T cells was determined. Arrows in the graph and amino acids F, S and Y indicate the stimulatory peptide termini.

In subject 411 (DRB1*03:01 DR1*09:01), HIVconsv vaccination induced CD4^+^ T cells recognizing HC080. One-residue truncated peptide FRKYTAFTIPSINN (FN14) provided the strongest signal, while shorter peptides were stimulatory down to the GFRKYTAFTIPS (GS12) and YTAFTIPSINN (YN11) (Figure 6). LANL-HSD lists FRKYTAFTIPSINN**E** as containing a DR supermotif.

#### HC081 YTAFTIPSINNETPG (Pol 282–296)

**Figure 7:**
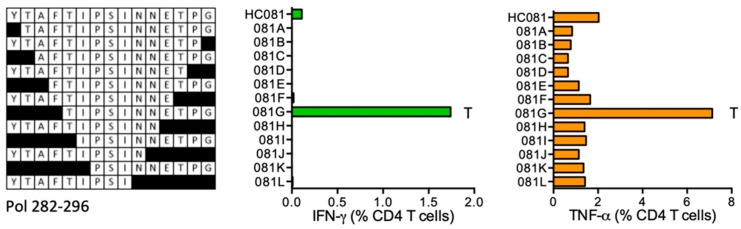
HC081/VID 415—definition of CD4^+^ T-cell epitopes in HIV-1 protein Pol. HC081 expanded PBMC from vaccine recipient 415 were tested in an ICS assay against serially truncated peptides and CD4^+^ T cells producing the IFN-γ (green) and TNF-α (orange) cytokines were enumerated using flow cytometry. Amino acid T indicates the stimulatory peptide terminus.

HC081 STCL of volunteer 415 (DRB1*11:04 DRB1*15:01) had detectable CD4^+^ T-cell responses to the HC081 peptide, but showed a much stronger response to shorter peptide TIPSINNETPG (TG11) consistent between both IFN-γ and TNF-α production (Figure 7). This has not been reported before and is not predicted to bind the participant’s DRB1 molecules.

#### HC088 GSPAIFQSSMTKILE- Pol (311–325)

**Figure 8:**
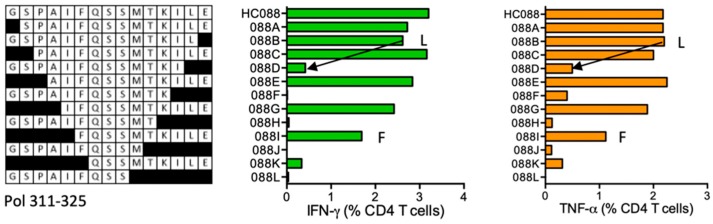
HC088/VID 411—definition of CD4^+^ T-cell epitopes in HIV-1 protein Pol. HC088 STCL of subject 411 was tested in an ICS assay against serially truncated peptides for induction IFN-γ (green) and TNF-α (orange) by CD4^+^ T cells. Arrows and amino acids F and L indicate the stimulatory peptide termini.

Volunteer 411 (DRB1*03:01 DR1*09:01) responded to an HIVconsv-derived peptide HC088, which was reported in the LANL-HSD without HLA restriction. Shorter peptides GSPAIFQSSMTKIL (GL14) and down to FQSSMTKILE (FE10) were stimulatory. Proline-extended peptide SPAIFQSSMTKILE**P** is reported in the LANL-HSD to carry a DR supermotif. ELF associated HC088 with the subject’s DRB1*04:01 allele (Figure 8).

#### HC093 KNPEIVIYQYMDDLYV (Pol 330–344; K was added for solubility)

**Figure 9:**
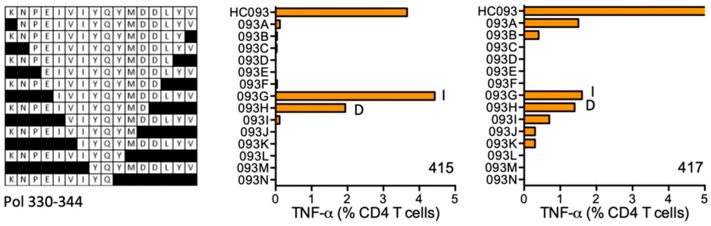
HC093/VID 415 and VID 417—definition of CD4^+^ T-cell epitopes in HIV-1 protein Pol. HC093-expanded PBMCs from subjects 415 and 417 were tested in ICS against shorter peptides (left) and CD4^+^ T cells producing TNF-α (orange) were enumerated. Amino acids I and D indicate the stimulatory peptide termini.

The CD4^+^ T-cell responses producing TNF-α were mounted to the Pol HC093 region by volunteers 415 (DRB1*11:04 DRB1*15:01) and 417 (DRB1*01:01 DRB1*07:01). HC093 STCL grown from both subjects responded to shorter peptides IVIYQYMDDLYV (IY12) and KNPEIVIYQYMD, however, for the latter peptide, the binding might be dependent on the N-terminal K, which was added to improve peptide solubility and, therefore, is not of HIV-1 origin (Figure 9). Peptide core IVIYQYM is predicted to bind DRB1*15:01.

#### HC094 VIYQYMDDLYVGSDL (Pol 334–348)

**Figure 10:**
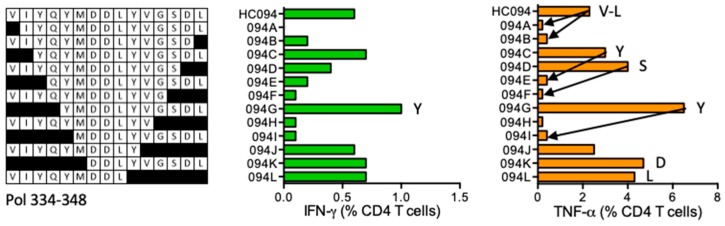
HC094/VID 420—definition of CD4^+^ T-cell epitopes in HIV-1 protein Pol. HC094 peptide-grown SCTL from volunteer 420 was stimulated with truncated peptides (left) in an ICS assay and production of IFN-γ (green) and TNF-α (orange) by CD4^+^ T cells was monitored. Arrows in the graph and amino acids V, L, Y, S and D indicate the stimulatory peptide termini.

HIVconsv vaccine recipient 420 (DRB1*11:01 DRB1*15:01) developed TNF-α dominated CD4^+^ T cell response to the HC094 peptide stimulation (Figure 10). HC094 STCL identified shorter stimulatory peptides likely covering multiple epitopes, which included VIYQYMDDLYVGS (VS13), VIYQYMDDL (YL9), YQYMDDLYVGSDL (YL13), YMDDLYVGSDL (YL11), and DDLYVGSDL (DL9). CD4^+^ T-cell epitopes in this Pol region have not been previously reported and DRB1*11:01 is the predicted restricting allele for the HC094 peptide.

#### HC102 KQVDRMRIRTWKSLVK (Vif 12–26; K was added for solubility)

**Figure 11:**
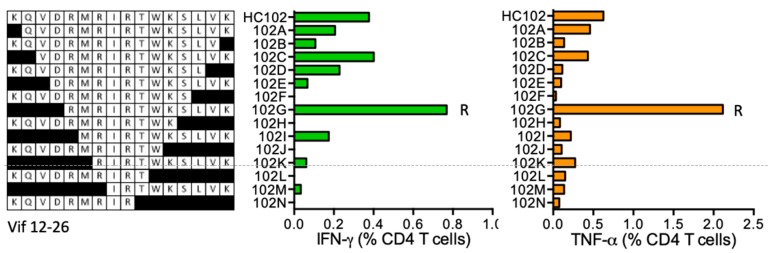
HC102/VID 403—definition of CD4^+^ T-cell epitopes in HIV-1 protein Vif. HC102 peptide-expanded SCTL from volunteer 403 were stimulated in an ICS assay using truncated peptides (left) and CD4^+^ T cells producing IFN-γ (green) and TNF-α (orange) were enumerated using flow cytometry. Amino acid R indicates the stimulatory peptide termini.

Subject 403 (DRB1*07:01 DRB1*16:01) responded to peptide HC102 and narrowing shorter optimal peptides stimulatory for CD4^+^ T cells revealed epitope RMRIRTWKSLVK (RK12) (Figure 11). Epitope core IRTWKS binding to the participant’s DRB1*07:01 molecule is predicted by ELF.

#### HC103 MRIRTWKSLVKHH-LT (Vif 16–28; ‘-’ indicates junction between two adjacent regions)

**Figure 12:**
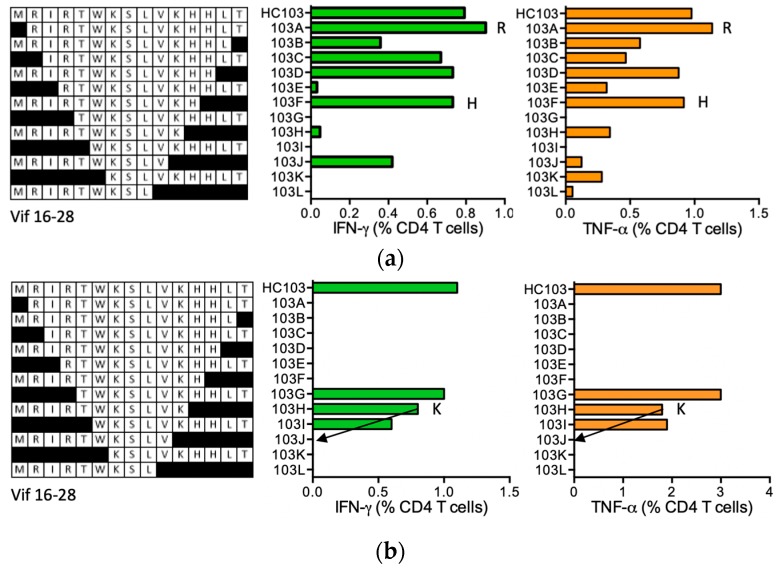
HC103/VIDs 406 and 409—definition of CD4^+^ T-cell epitopes in HIV-1 protein Vif. HC103 STCL of vaccine recipients 406 (**a**) and 409 (**b**) were incubated with shorter peptides (left) and the production of IFN-γ (green) and TNF-α (orange) by CD4^+^ T cells was quantified in a polychromatic flow cytometry. Arrows and amino acid K indicate the stimulatory peptide termini.

HC103 is a junctional peptide spanning two regions made adjacent by the immunogen chimeric design with the border indicated by a hyphen, of which the biggest part was derived from Vif. For this Vif region, no epitopes are recorded in the LANL-HSD. Two HIVconsv vaccine recipients made CD4^+^ T-cell responses to HC103. HC103 STCL of volunteer 406 (DRB1*01:01 DRB1*07:01) recognized shorter peptides MRIRTWKSLVKHH (MH13) and MRIRTWKSLVKH (MH12) (Figure 12a), which are predicted to bind DRB1*07:01. Peptide RIRTWKSLVKHH-LT (RT14) truncated at the N-terminus provided a moderately stronger stimulus than parental HC103. Volunteer 409 (DRB1*03:01 DRB1*15:01) had the strongest IFN-γ responses of the two HC103 responders. The only stimulatory, fully HIV-1-derived shorter epitope was MRIRTWKSLVK (MK10) (Figure 12b), for which epitope core IRTWKSLV was predicted to bind to both the DRB1*03:01 and *15:01 molecules.

#### HC115 KQ-YWQATWIPEWEFVN (Pol 560–573; K was added for solubility; ‘-’ indicates junction between two adjacent regions)

**Figure 13:**
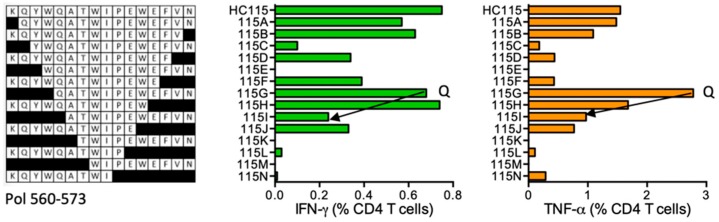
HC115/VID 414—definition of CD4^+^ T-cell epitopes in HIV-1 protein Pol. HC115-grown SCTL from subject 414 was stimulated with progressively truncated peptides (left) in an ICS assay and production of IFN-γ (green) and TNF-α (orange) by CD4^+^ T cells was measured by flow cytometry. Arrows in the graph and amino acid Q indicate the stimulatory peptide termini.

HIVconsv vaccination primed CD4^+^ T-cell responses in subject 414 (DRB1*01:02 DRB1*04:01) to epitopes in HC115, in which K was added to improve peptide solubility. Narrowing the length identified peptides QATWIPEWEFVN (QN12) and non-HIV-1 KQYWQATWIPEW (KW12) (Figure 13). QN12 is not reported in the LANL-HSD and is not predicted by ELF.

#### HC123 KNFNMWKNDMVDQMHE (gp160 91–106; K was added for solubility)

**Figure 14:**
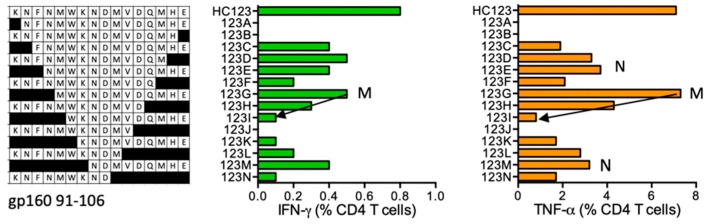
HC123/VID 420—definition of CD4^+^ T-cell epitopes in HIV-1 protein glycoprotein gp160. HC123-expanded PBMCs from subject 420 were tested in ICS against shorter peptides (left) and CD4^+^ T cells producing IFN-γ (green) and TNF-α (orange) were enumerated. Arrows and amino acids M and N indicate the stimulatory peptide termini.

Volunteer 420 (DRB1*11:04 DRB1*15:01) responded to HC123. The strongest, shorter, stimulatory, HIV-1-derived (i.e., without the N-terminal K) peptides were FNMWKNDMVDQMHE (NE14), NMWKNDMVDQMHE (NE13), MWKNDMVDQMHE (ME12), KNDMVDQMHE (KE10), and NDMVDQMHE (NE9) stimulating mainly TNF-α production (Figure 14). Variant peptide **NVTE**NFNMWKN**N**MV**E**QMH is reported in the LANL-HSD to bind both subjects’ DRB1 molecules.

#### HC145 GQVDCSPGIWQLDCTH (Pol 767–782)

**Figure 15:**
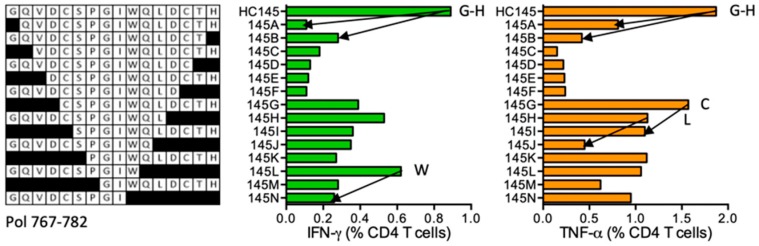
HC145/VID 404—definition of CD4^+^ T-cell epitopes in HIV-1 protein Pol. HC145-expanded SCTL of volunteer 404 was stimulated in an ICS assay using truncated peptides (left) and CD4^+^ T cell frequencies producing IFN-γ (green) and TNF-α (orange) were determined using flow cytometry. Arrows and amino acids G, H, W, C and L indicate the stimulatory peptide termini.

Subject 404 (DRB1*13:01 DRB1*13:02) developed CD4^+^ T-cell responses specific for HC145. The ICS assay confirmed the GH15 reactivity and GQVDCSPGIWQL (GL12), GQVDCSPGIW (GW10), CSPGIWQLDCTH (CH12), and SPGIWQLDCTH (H11) to be shorter stimulatory epitopes (Figure 15). Epitopes in this Pol region are not listed in the LANL-HSD and no prediction for restriction is suggested by ELF.

#### HC164 VQMAVFIHNFKRKGGI (Pol 891–906)

**Figure 16:**
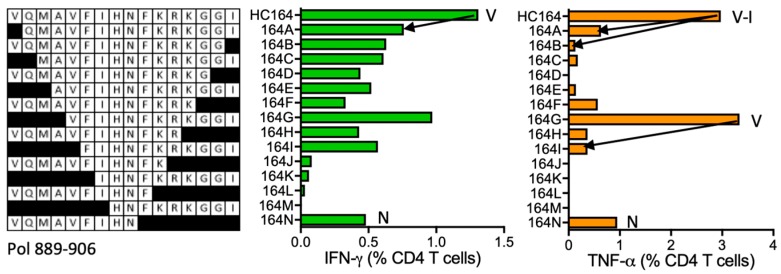
HC164/VID 404—definition of CD4^+^ T-cell epitopes in HIV-1 protein Pol. Subject 404′s PBMCs were expanded in culture using HC164 stimulation and the resulting STCL was tested using shorter peptide derivatives (left) for IFN-γ (green) and TNF-α (orange) production. Arrows and amino acids V, N and I indicate the stimulatory peptide termini.

HC164 peptide was stimulatory for CD4^+^ T cells from subject 404 (DRB1*13:01 DRB1*13:02). The ICS assay confirmed peptide HC164 to be stimulatory and also detected stimulation by VQMAVFIHN (VN9) and VFIHNFKRKGGI (VI12) (Figure 16). **KTA**VQMAVFIHNFKR is reported to contain a DR-binding supermotif.

#### HC176 VVPRRKAKIIRDYGK (Pol 974–988)

**Figure 17:**
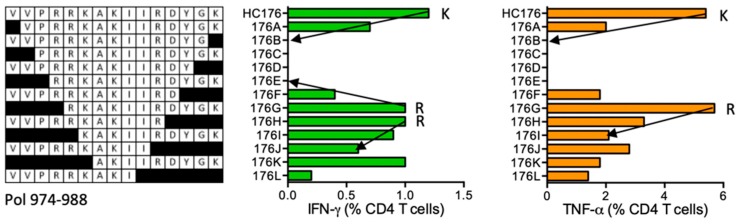
HC176/VID 413—definition of CD4^+^ T-cell epitopes in HIV-1 protein Pol. HC176-cultured STCL from subject 413 were tested in an ICS assay using truncated peptides (left) for IFN-γ (green) and TNF-α (orange) production using flow cytometry. Arrows and amino acids K and R indicate the stimulatory peptide termini.

Volunteer 413 (DRB1*03:01 DRB1*11:01) responded to peptide HC176. The ICS assay using HC176 STCL confirmed stimulation by the HC176 peptide and also VVPRRKAKIIR (VR11), VVPRRKAKII (VI10), VPRRKAKIIRDYGK (VK14), RKAKIIRDYGK (RK11), KAKIIRDYGK (KK10), and AKIIRDYGK (AK9) (Figure 17). Overlapping peptides **SDIK**VVPRRKAKIIR are reported in the LANL-HSD without restriction and only HC176 is predicted by ELF to bind DRB1*11:01.

#### HC177 RKAKIIRDYGKQMAG (Pol 978–992)

**Figure 18:**
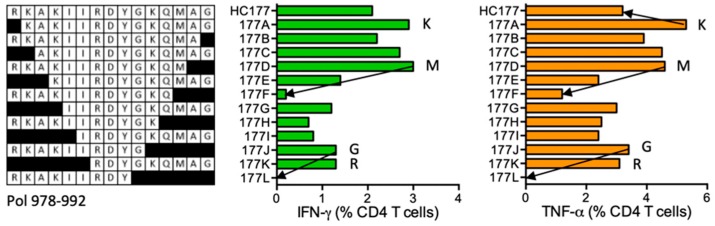
HC177/VID 409—definition of CD4^+^ T-cell epitopes in HIV-1 protein Pol. HC177 STCL generated from subject 409 were tested against shorter peptides (left) in the ICS assay for IFN-γ (green) and TNF-α (orange) production. Arrows and amino acids K, M, G and R indicate the stimulatory peptide termini.

Subject 409 (DRB1*03:01 DRB1*15:01) developed CD4^+^ T cells specific for HC177. A number of shorter stimulatory peptides were identified including RKAKIIRDYGKQMA (RA14), RKAKIIRDYGKQM (RM13), and RKAKIIRDYG (RG10), and from KAKIIRDYGKQMAG (KG14) down to RDYGKQMAG (RG9) (Figure 18). No epitopes were listed in the LANL-HSD for this Pol region. IIRDYGKQM is a predicted peptide core binding to DRB1*03:01.

#### HC178 IIRDYGKQMAGADCV (Pol 982–996)

**Figure 19:**
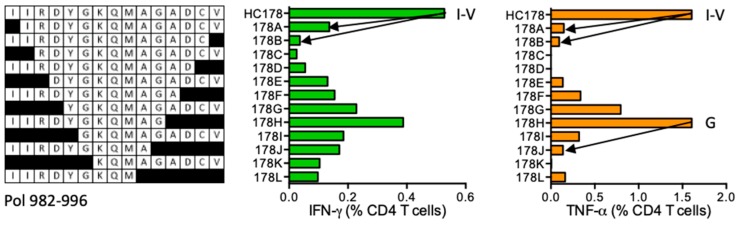
HC178/VID 403—definition of CD4^+^ T-cell epitopes in HIV-1 protein Pol. HC178 STCLs from subject 403 were tested against shorter peptides (left) in the ICS assay for IFN-γ (green) and TNF-α (orange) production. Arrows and amino acids I, V and G indicate the stimulatory peptide termini. Arrows and amino acids I, V and G indicate the stimulatory peptide termini.

Subject 403 (DRB1*07:01 DRB1*16:01) developed CD4^+^ T cells specific for HC178 and the ICS assay using participant´s HC178 STCL confirmed peptide IIRDYGKQMAGADCV (IV15) and peptide IIRDYGKQMAG (IG11) (Figure 19). No epitopes are reported or predicted in this sequence.

#### HC190 LLRAIEAQQHLLQLT (gp160 746–760)

**Figure 20:**
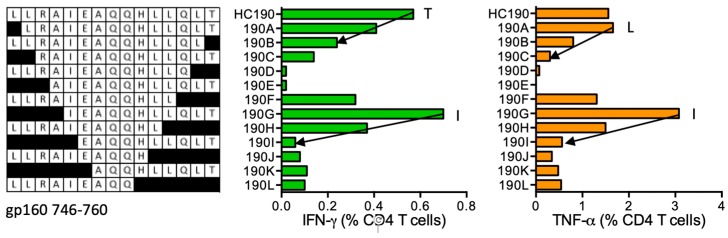
HC190/VID 414—definition of CD4^+^ T-cell epitopes in HIV-1 glycoprotein gp160. HC190 peptide-expanded PBMCs of VID 414 were incubated with truncated peptides (left) and frequencies of CD4^+^ T cells producing IFN-γ (green) and TNF-α (orange) were determined using polychromatic flow cytometry. Arrows and amino acids T, I and L indicate the stimulatory peptide termini.

HIVconsv vaccine recipient 414 (DRB1*01:02 DRB1*04:01) responded to HC190 and recognized the shorter peptides LLRAIEAQQHLL (LL12), LLRAIEAQQHL (LL11), LRAIEAQQHLLQLT (LT14), and IEAQQHLLQLT (IT11) (Figure 20), which are not reported as HLA class II binding epitopes. HC190 was predicted to bind DRB1*01:02, while the binding preference for peptide core LLRAIEAQQ is predicted for DRB1*04:01.

All CD4^+^ T cell epitope mapping data are summarized in Table 2.

## 4. Discussion

In the course of this work, we have defined novel peptides stimulatory for CD4^+^ T cells elicited by a candidate HIV-1 vaccine in healthy, HIV-1/2-negative volunteers in Oxford, UK. This vaccine delivered the first-generation conserved regions of HIV-1 proteins employing clade consensus amino acid sequences, which were assembled into a chimeric immunogen designated HIVconsv [40]. Both the use of conserved regions frequently subdominant in natural infection and utilization of potentially artificial consensus sequences might have contributed to the relatively high discovery rate of novel, previously unreported epitopes absent from the LANL-HSD. In contrast, reported CD4^+^ T-cell epitopes reported in Gag p24 EEKAFSPEV (Gag 160–168) and EKAFSPEVIPMFSAL (Gag 161–175) restricted by DRB1*01:01 were present in the HIVconsv immunogen (peptide HC001), but were not immunogenic even in volunteers expressing the correct restricting DRB1 allele. This may be due to the epitope location at the very N-terminus of the HIVconsv protein, only preceded by the translational start methionine, which might have interfered with peptide processing or led to epitope destruction [48,49,50].

Our results concur with previous studies in that each STCL expanded in vitro by a 10-day culture with a 15-mer peptide recognized one or multiple clusters of shorter peptides likely each centered around a peptide core, the interaction of which with the HLA class II groove is usually characterized by a motif of anchor residues [5,6,31,51,52]. However, it remained challenging to define the ‘optimal’ binding/stimulatory peptide length as the strongest stimulatory peptides were often 11 or 12 residues long, yet sometimes shorter versions clearly induced robust reactivities, too. The 11/12-amino acid lengths may be optimal by the virtue of the flanking region sequences for both binding to the restricting HLA class II molecules and interacting with the T-cell receptors on these CD4^+^ cells as documented by the near 60 MHC class II structures. Despite this increasing amount of structural information, there is no simple solution to the ‘optimal epitope length’ conundrum. While optimal peptide length may exist for a combination of defined HLA class II allele with a particular T-cell receptor, it is not likely to be the minimal stimulatory peptide length. Thus, a class II epitope may be best described collectively by definition of the longest and shortest (may be shorter than core) peptide length of nested stimulatory peptides, identification of the binding epitope core guided by a binding motif and determination of the strongest stimulatory peptide length. Although much more stringent, similar plasticity is emerging for the definition of HLA class I epitopes [17], but it is likely much more pronounced for class II epitopes given the open ends of the peptide-binding cleft. Bearing in mind that all stimulatory lengths may contribute to and some modulate negatively the immune response [33,34,35,36,37,38], these parameters may vary depending on the readout: production of IFN-γ, TNF-α, IL-2, or cytotoxicity, and so forth, and that multiple epitopes may be overlapping. For these reasons, in Table 2, we listed all tested peptides stimulatory for at least 1% of expanded CD4^+^ T-cell lines and indicated the relative balance in the production of the two measured cytokines.

HIV-1-specific CD4^+^ T cells display multiple functions [5,6,31,51]. In the present study, CD4^+^ T cells were capable of proliferation and production of at least IFN-γ and TNF-α, two important antiviral and proinflammatory cytokines. Bearing in mind in vitro expansion, these two functions were not always performed by equal frequencies of responding cells. What governs these quantitative and qualitative differences remains unclear, but the presentation of the HIVconsv immunogen to the immune system, here by the combination of intramuscular DNA, nonreplicating simian adenovirus, and MVA delivery, likely critically impacted on the type of induced responses by analogy to different viruses or bacteria inducing different types of immune memory [1,7,53,54]. Thus, for an efficacious vaccine, at the very least the immunogens and means of their delivery will need to be optimized to maximize induction of protective T-cell traits [1,54,55]. For HIV-1, this may also include success at inducing T-cell populations with access to B-cell follicles, the sites of persistent HIV-1 reservoir and replication [56]. As for epitope mapping, some responses may be easier/more difficult to detect by one or the other cytokine and the stimulation facilitates/complicates precise epitope mapping. Also, the interaction of the CD4 coreceptor with MHC class II greatly reduces the number of HLA-TCR engagements required for T-cell activation [57] and substantially increases cytokine production by CD4^+^ T cells [58], and this interaction may vary for different responses.

A previous study, which mapped comprehensively CD4^+^ T-cell responses across the whole proteome in HIV-1-positive individuals, identified immunodominant responses to the Gag and Nef proteins [31]. This was not the case following HIVconsv vaccination, which induced prominent Pol epitope responses directly proportional to the relative length of the Pol segment in the HIVconsv sequence (Table 3). These results would support the ability of a potent vector delivery to (re)focus immune responses including CD4^+^ T cells toward regions that are generally subdominant in natural HIV-1 infection [59,60]. A different study reported over half of tested variant peptides not being recognized by CD4^+^ T cells [51], emphasizing even for the more promiscuous HLA class II molecules peptide selectivity and the importance of focusing on and matching vaccine responses to less variable regions of the HIV-1 proteome. Finally, 11 helper T-cell peptides with HLA-DR supermotif, which were 82% conserved in clade B isolates, were identified and their stimulatory ability were confirmed in HIV-1-positive patients [21].

One of the critical CD4^+^ T-cell traits for achieving protection against HIV-1 is T-cell frequency. The combination of intramuscularly delivered simian adenovirus prime and MVA boost (with or without prior DNA) is on the forefront of currently tested regimens of nonreplicating vaccine vectors in humans [44,61,62,63]. In the present work, we did not evaluate the true frequencies of CD4^+^ T-cell responses with individual specificities in peripheral blood, but rather focused on their specificities, which was greatly facilitated by the use of in vitro expanded T-cell cultures. We were also unable to unequivocally identify the HLA class II restricting molecules beyond the tissue typing of vaccine recipients and performing binding predictions using the ELF algorithm. Finally, it would have been interesting to investigate the clonality of the CD4^+^ T cells responding to individual peptide clusters or whether CD4^+^ T cells display generally higher or lower clonality compared to CD8^+^ T cells. Regardless of these limitations, our effort contributes to the collection of CD4^+^ T-cell determinants in HIV-1, which will drive iterative vaccine improvements especially in the light of the increasingly recognized important roles of CD4^+^ T cells in the generation of protective anti-HIV-1 responses.

## Figures and Tables

**Table 1:** HIV-CORE 002 vaccine recipients, tissue types, regimens, and peak IFN-γ ELISPOT assay responses induced by the HIVconsv vaccines.

Volunteer ID	HLA Class II			Regimen ^1^	Peak IFN-γ ELISPOT
	DRB1	DRB2/3/4	DQB1		(SFU/10^6^ PBMC) ^2^
403	*07:01 *16:01	DRB4 DRB5	02 05	CM	1475
404	*13:01 *13:02	DRB3 DRB3	*06:02 *06:05	CM	3780
406	*01:01 *07:01	DRB4 DRB4	02 05	CM	5170
409	*03:01 *15:01	DRB3 DRB5	02 *06:02	CM	5150
411	*03:01 *09:01	BRB3 DRB5	02 *03:03	CM	12,020
413	*03:01 *11:01	DRB3 DRB3	02 *03:01	DDDCM	6240
414	*01:02 *04:01	DRB4 DRB4	05 *03:01	DDDCM	7960
415	*11:04 *15:01	DRB3 DRB5	*06:02 *03:01	DDDCM	5340
417	*01:01 *07:01	BRB4 DRB4	02 05	DDDCM	4260
418	*04:01 *07:01	DRB4 DRB4	*03:01 *03:03	DDDCM	7060
420	*11:01 *15:01	DRB3 DRB5	*03:01 *06:02	DDDCM	1530

^1^ D—plasmid DNA pSG2.HIVconsv 4 mg; C—ChAdV63.HIVconsv 5 × 10^10^ virus particles; M—MVA.HIVconsv. 2 × 10^8^ plaque-forming units. All vaccines were delivered intramuscularly. ^2^ HIV-specific spot-forming units (can be a cluster of cells) per one million of peripheral mononuclear cells.

**Table 2:** Conserved HIV-1-derived HLA-DRB1-restricted epitopes recognized by CD4^+^ T cells.

Peptide No.	Protein (HXB2 Position)	Stimulatory Peptides	IFN-γ/TNF-α	Responder’s HLA-DRB1	Predicted HLA-DRB1	Reported Epitopes	Reported Epitopes’ Restriction	Epitope Core	Epitope Core HLA-DRB1
HC003	Gag (167–181)	EVIPMFTALSEGATP EVIPMFTALSEGAT EVIPMFTALSEGA EVIPMFTALSEG EVIPMFTALSE EVIPMFTALS	IFN-γ ≈ TNF-α	*04:01 *07:01	*04:01 *07:01	PMFTALSEGAT **P**EVIPMF**S**ALSEGA EVIPMF**S**ALS	Human DR1 *01:01 DR4	EVIPMFTALS FTALSEGAT VIPMFT LSEGAT	*04:01 *04:01 *07:01 *07:01
HC017	Gag (269–283)	GLNKIVRMYSPVSIL GLNKIVRMYSPVSI GLNKIVRMYSPVS	IFN-γ > TNF-α	*04:01 *07:01	*04:01 *07:01	GLNKIVRMYSPVSIL GLNKIVRMYSP**T**SIL LNKIVRMYSPVSILD LNKIVRMYSP**T**SILD KIVRMYSP**T**S KIVRMYSP**T**	human *01:01 human human DR4 *01:01	LNKIVRMYS IVRMYS	*04:01 *07:01
		LNKIVRMYSPVSIL NKIVRMYSPVSIL KIVRMYSPVSIL							
HC078	Pol (270–284)	YFSVPLDEGFRKYTA FSVPLDEGFRKYTA SVPLDEGFRKYTA VPLDEGFRKYTA PLDEGFRKYTA	IFN-γ > TNF-α IFN-γ TNF-α	*01:01 *07:01					
HC079	Pol (274–288)	PLDEGFRKYTAFTIP FRKYTAFTIP	TNF-α IFN-γ	*01:01 *07:01					
HC080	Pol (278–292)	GFRKYTAFTIPSINN GFRKYTAFTIPSIN GFRKYTAFTIPSI GFRKYTAFTIPS	IFN-γ ≈ TNF-α	*03:01 *09:01	*07:01	FRKYTAFTIPSINNE	DR supermotif		
		FRKYTAFTIPSINN RKYTAFTIPSINN KYTAFTIPSINN YTAFTIPSINN							
HC081	Pol (282–296)	YTAFTIPSINNETPG TIPSINNETPG	TNF-α IFN-γ < TNF-α	*11:04 *15:01					
HC088	Pol (311–325)	GSPAIFQSSMTKILE. GSPAIFQSSMTKIL	IFN-γ ≈ TNF-α	*03:01 *09:01	*04:01	GSPAIFQSSMTKILE SPAIFQSSMTKILEP	human DR supermotif		
		SPAIFQSSMTKILE PAIFQSSMTKILE AIFQSSMTKILE IFQSSMTKILE FQSSMTKILE							
HC093	Pol (330–344)	**K**NPEIVIYQYMDDLYV **K**NPEIVIYQYMD NPEIVIYQYMDDLYV IVIYQYMDDLYV	TNF-α TNF-α	*11:04 *15:01 *01:01 *07:01				IVIYQYM	*15:01
HC094	Pol (334–348)	VIYQYMDDLYVGSDL VIYQYMDDLYVGS VIYQYMDDL	IFN-γ < TNF-α	*11:01 *15:01	*11:01				
		YQYMDDLYVGSDL YMDDLYVGSDL DDLYVGSDL							
HC102	Vif (12–26)	----RMRIRTWKSLVK	IFN-γ < TNF-α	*07:01 *16:01	*07:01			IRTWKS	*07:01
HC103	Vif (16–28)	MRIRTWKSLVKHH-LT MRIRTWKSLVKHH MRIRTWKSLVKH RIRTWKSLVKHH-LT	IFN-γ < TNF-α	*07:01 *16:01					*07:01
HC103	Vif (16–28)	MRIRTWKSLVKHH-LT MRIRTWKSLVK	IFN-γ < TNF-α	*03:01 *15:01	*03:01 *15:01			IRTWKSLV	*03:01 *15:01
HC115	Pol (560–573)	KQ-YWQATWIPEWEFVN QATWIPEWEFVN	IFN-γ < TNF-α	*01:02 *04:01					
HC123	gp160 (91–106)	KNFNMWKNDMVDQMHE FNMWKNDMVDQMHE NMWKNDMVDQMHE MWKNDMVDQMHE NDMVDQMHE	IFN-γ < TNF-α TNF-α	*11:01 *15:01		NVTENFNMWKNNMVEQMH	*11:01 *15:01 *15:02		
HC145	Pol (767–782)	GQVDCSPGIWQLDCTH GQVDCSPGIWQL GQVDCSPGIW CSPGIWQLDCTH SPGIWQLDCTH	IFN-γ ≈ TNF-α	*13:01 *13:02					
HC164	Pol (891–906)	VQMAVFIHNFKRKGGI VQMAVFIHN VFIHNFKRKGGI	IFN-γ < TNF-α	*13:01 *13:02	*13:01	KTAVQMAVFIHNFKR	DR supermotif		
HC176	Pol (974–988)	VVPRRKAKIIRDYGK VVPRRKAKIIR VVPRRKAKII	IFN-γ < TNF-α	*03:01 *11:01	*11:01	SDIKVVPRRKAKIIR	human		
		VPRRKAKIIRDYGK RKAKIIRDYGK KAKIIRDYGK AKIIRDYGK							
HC177	Pol (978–992)	RKAKIIRDYGKQMAG RKAKIIRDYGKQMA RKAKIIRDYGKQM RKAKIIRDYG	IFN-γ ≈ TNF-α	*03:01 *15:01	*03:01			IIRDYGKQM	*03:01
		KAKIIRDYGKQMAG AKIIRDYGKQMAG KIIRDYGKQMAG IIRDYGKQMAG IRDYGKQMAG RDYGKQMAG							
HC178	Pol (982–996)	IIRDYGKQMAGADCV IIRDYGKQMAG	IFN-γ < TNF-α	*07:01 *16:01					
HC190	gp160 (745–760)	LLRAIEAQQHLLQLT LLRAIEAQQHLL LLRAIEAQQHL LLRAIEAQQHLLQLT IEAQQHLLQLT		*01:02 *04:01	*01:02			LLRAIEAQQ	*04:01

**Table 3:** Correlation between stimulatory CD4^+^ T-cell epitopes and HIVconsv protein proportion.

Protein	Total Region Length (AA Residues)	Stimulatory CD4 T-Cell Epitopes (No.)
Gag	134 (17%)	2 (11%)
Pol	526 (68%)	13 (68%)
Vif	28 (4%)	2 (11%)
Env	91 (12%)	2 (11%)
HIVconsv	776 (100%)	19 (100%)
